# Transient Silencing of a Type IV P-Type ATPase, *Atp10c*, Results in Decreased Glucose Uptake in C2C12 Myotubes

**DOI:** 10.1155/2012/152902

**Published:** 2012-02-29

**Authors:** S. E. Hurst, S. C. Minkin, J. Biggerstaff, M. S. Dhar

**Affiliations:** ^1^Comparative and Experimental Medicine, College of Veterinary Medicine, University of Tennessee, Knoxville, TN 37996, USA; ^2^Department of Large Animal Clinical Sciences, College of Veterinary Medicine, University of Tennessee, Knoxville, TN 37996, USA; ^3^Center for Environmental Biotechnology, University of Tennessee, Knoxville, TN 37996, USA

## Abstract

*Atp10c* is a strong candidate gene for diet-induced obesity and type 2 diabetes. To identify molecular and cellular targets of ATP10C, *Atp10c* expression was altered *in vitro* in C2C12 skeletal muscle myotubes by transient transfection with an *Atp10c*-specific siRNA. Glucose uptake assays revealed that insulin stimulation caused a significant 2.54-fold decrease in 2-deoxyglucose uptake in transfected cells coupled with a significant upregulation of native mitogen-activated protein kinases (MAPKs), p38, and p44/42. Additionally, glucose transporter-1 (GLUT1) was significantly upregulated; no changes in glucose transporter-4 (GLUT4) expression were observed. The involvement of MAPKs was confirmed using the specific inhibitor SB203580, which downregulated the expression of native and phosphorylated MAPK proteins in transfected cells without any changes in insulin-stimulated glucose uptake. Results indicate that *Atp10c* regulates glucose metabolism, at least in part via the MAPK pathway, and, thus, plays a significant role in the development of insulin resistance and type 2 diabetes.

## 1. Introduction

In humans, skeletal muscle accounts for nearly 40% of the body's mass and serves as the main tissue involved in glucose uptake during insulin stimulation. Several researchers have established that glucose consumption in skeletal muscle decreases with type 2 diabetes mellitus. This reduced glucose consumption is a result of impaired transduction of insulin signals, such as insulin receptor substrate-1 phosphorylation, phosphatidylinositol 3-kinase (PI3K) activity, mitogen-activated protein kinase (MAPK) activity, insulin-responsive glucose transporters, namely, glucose transporter-4 (GLUT4), and/or other insulin-independent mechanisms [1]. Therefore, a detailed analysis of insulin signaling at the cellular and molecular level is critical to understand the pathogenesis of type 2 diabetes associated with obesity.

Heterozygous *Atp10c* mice present with the disease states of insulin resistance and obesity, as well as a host of other related disorders, including hyperlipidemia and hyperinsulinemia. Previous research using these mice indicates that the *Atp10c *gene appears to be a strong candidate gene for diet-induced obesity and type 2 diabetes mellitus [2]. *Atp10c* is a putative phospholipid translocase or “flippase,” which encodes for a type IV P-type ATPase. *Atp10c *maps to the *p*-locus on mouse chromosome 7, to a region of a quantitative trait locus associated with body weight, body fat, and diabetic phenotypes. The human ortholog, ATP10C, maps to the syntenic region on chromosome *15q12* and is also associated with an elevated body mass index [3, 4]. Moreover, microarray gene profiling on *Atp10c* heterozygous mice indicated significant changes in the mRNA expression of factors involved in insulin-dependent and insulin-independent glucose uptake [5].

Although flippases, like *Atp10c*, have been studied for many years, their exact character and function remain unclear. These proteins are believed to maintain the asymmetry of the lipid bilayer by translocating specific phospholipids from one leaflet to the other and vice versa [6], but they may also participate in the formation of transport vesicles [7]. Moreover, deficiencies in these proteins have been shown to cause defects in lipid metabolism and have been implicated in the disease states of obesity, type 2 diabetes, and nonalcoholic fatty liver disease [2]. Not much is known about the role of ATP10C in regulating insulin resistance in skeletal muscle, if any, and its possible molecular and cellular targets have not been investigated.

In view of the above literature, we hypothesized that the type IV P-type ATPase, ATP10C, has an important role in glucose metabolism. Since ATP10C is a transmembrane protein it might exert its effect via multiple signaling pathways: (1) acting solely at the plasma membrane to maintain the nonrandom distribution of phospholipids, thus contributing to a proper membrane environment for normal protein sequestration and function, (2) acting at the plasma membrane affecting the biogenesis of membrane vesicles important for plasma membrane delivery and/or retrieval of glucose transporter proteins in basal and insulin-stimulated states, and (3) acting directly on the expression, translocation, and/or function of glucose transporter proteins themselves. Since the MAPK pathway is known to be a key signaling cascade which mediates glucose clearance/uptake by the skeletal muscle in presence or absence of insulin, we tested whether the MAPKs, in general, are the targets of ATP10C. To prove our hypothesis, specific objectives were to (a) establish a tissue culture system of mouse skeletal muscle wherein endogenous expression of *Atp10c* could be monitored, (b) alter the endogenous level of *Atp10c* expression by siRNA, and (c) measure glucose uptake and assess changes in expression of MAPKs involved in this process.

## 2. Materials and Methods

### 2.1. Materials

Mouse skeletal muscle cell line C2C12, a commercially available cell line, was kindly provided by Dr. Seung Baek, College of Veterinary Medicine, the University of Tennessee, Knoxville, TN, USA. Dulbecco's modified Eagle medium (DMEM) containing 4.5 mg/L glucose and 4.5 mM/L L-glutamine, antibiotics (100 IU/mL penicillin and 100 *μ*g/mL streptomycin), ABsolute Blue SYBR Green ROX quantitative PCR mix, bovine calf serum (BCS), and radioimmunoprecipitation assay (RIPA) buffer were from Thermo Fisher Scientific (Waltham, MA). DMEM with 1% antibiotics and 10% BCS is furthermore referred to as the complete growth media. Horse serum, 2-deoxy [^3^H] glucose (2-DOG), protease inhibitor cocktail in DMSO solution, MAPK inhibitor SB203580, and human insulin solution (10 mg/mL in HEPES, pH 8.2) were from Sigma Aldrich (St. Louis, MO). A bicinchoninic acid kit (BCA) and an enhanced chemiluminescence (ECL) western blotting Detection Kit were purchased from Pierce Biotech Inc. (Rockford, IL) and used in protein experiments. Primary antibodies (p38, phospho-p38, JNK, phospho-JNK, p44/42, and phospho-p44/42) as well as the secondary antibody, horseradish peroxidase- (HRP-) conjugated anti-rabbit IgG, were obtained from Cell Signaling Technology (Danvers, MA). Caveolin-1 was used as an immunoblot control and was purchased from Santa Cruz Biotechnologies (Santa Cruz, CA). The secondary antibody, HRP-conjugated anti-goat IgG, was also obtained from Santa Cruz Biotechnologies. HiPerfect transfection reagent, *Atp10c*-specific siRNA constructs, RNeasy Mini Kit, and QuantiTect primer assays for *MyoD *and *Atp10c* were from Qiagen (Valencia, CA). Quantitative PCR primers specific for mouse glyceraldehyde 3-phosphate dehydrogenase (*Gapdh*) were designed using the Primer 3 program (http://primer3.sourceforge.net/) and were commercially obtained from Operon (Huntsville, AL). The iScript cDNA synthesis kit was acquired from Bio-Rad Laboratories (Hercules, CA). Anti-GLUT1 and GLUT4 were kindly provided by Dr. Samuel Cushman, National Institute of Diabetes and Digestive and Kidney Diseases, National Institutes of Health, Bethesda, MD. All immunofluorescence materials (protein blocks [normal rabbit], negative control [normal rabbit], and antibody diluent) were purchased from BioGenex (San Ramon, CA). Millicell EZ slides from Millipore (Billerica, MA) were used for immunofluorescence experiments. Secondary antibody specific for immunofluorescence application, Alexa Fluor 568 donkey anti-rabbit, as well as Prolong Gold Antifade reagent was purchased from Invitrogen (Carlsbad, CA).

### 2.2. Cell Culture and Treatments

C2C12 myoblasts were cultured as described elsewhere [8, 9]. Roughly 2.0 × 10^5^ cells were seeded in a 60 mm dish or a single well of a 6-well plate. They were maintained at 37°C and 5% CO_2_ in complete growth media. Cells at 70% confluency were differentiated in the presence of 2% horse serum-enriched media for 3–5 days. Completely differentiated myotubes (days 3–5) were either subjected to various treatments described in the relevant sections or harvested for subsequent experiments.

### 2.3. siRNA Transfection

Three different siRNA oligonucleotides against *Atp10c* were commercially obtained (Qiagen); one was generated from the sequence at the 3′ end of the *Atp10c *gene, SI00906220 (sense: r[CCU GGG UAU UGA AAC CAA A]dTdT and antisense: r[UUU GGU UUC AAU ACC CAG G]dTdG), and the second, SI00906213, and third, SI00906206, were generated from the sequence at the 5′ end (sense: r[CGU CUU UGC UGC AAU GAA A]dTdT and antisense: r[UUU CAU UGC AGC AAA GAC G]dGdA). C2C12 myotubes were transiently transfected with *Atp10c*-specific and scrambled siRNA using HiPerfect transfection reagent (Qiagen) according to the manufacturer's instructions. Myotubes transfected with siRNA were either harvested or treated with the reagents indicated in the relevant sections and/or used to measure glucose uptake.

In the first experiment, the optimum concentration and time of knockdown for each siRNA used were determined. Briefly, HiPerfect transfection regent and siRNAs were mixed at various concentrations (0, 50, 100, and 200 nM) to form a complex. The transfection complexes were then applied to designated cells and incubated for 24, 48, or 72 h before subsequent analysis. C2C12 myotubes demonstrating efficient *Atp10c* knockdown and, therefore, used in all further experiments were designated as C2*10c*/−. Mock-transfected (i.e., transfected with HiPerfect only) C2C12 myotubes (C2wt) were used as corresponding controls.

### 2.4. RNA, cDNA Synthesis, and Quantitative PCR

The following procedures were performed as described elsewhere [5, 10, 11]. Cells were washed with ice cold 1X PBS, and total RNAs were isolated using RNeasy Mini RNA kit (Qiagen), according to the manufacturer's instructions. Single-stranded cDNA was synthesized using the iScript cDNA synthesis kit (Bio-Rad) and amplified using gene-specific primers by quantitative PCR (qPCR by Quantitect primer assays), with mouse *Gapdh *as the housekeeping gene. All mRNA expressions were achieved by qPCR using ABsolute SYBR Green ROX quantitative PCR mix on the Strategene Mx3005P with MxPro analysis software under the following PCR conditions: 1 cycle of 50°C for 15 min and 95°C for 2 min, followed by 40 cycles of 95°C for 25 s, 52°C for 25 s, and 72°C for 1 min. The relative abundance of target gene expression was calculated using the 2-ΔΔCT and standard curve method, with ΔΔCT being the difference between CT of the target gene normalized with respect to the *Gapdh* CT [12].

### 2.5. Preparation of Cellular Extracts, Immunoblotting, and Immunofluorescence

Total cell lysates were isolated using RIPA buffer according to standard methods [13, 14]. Briefly, cells were washed twice with 1X PBS and lysed in RIPA buffer containing protease inhibitor cocktails at 4°C for 30 min. Lysates were centrifuged at 16,000 g for 10 min at 4°C. Protein estimation was performed using the BCA kit (Pierce Biotech), according to the manufacturer's instructions.

Immunoblot analysis was carried out according to standard procedures [13, 14]. Equal concentrations (25−100 *μ*gs) of proteins were resolved on 10% SDS-PAGE, using 5X Laemmli sample buffer containing Tris-HCL (375 mM, pH 6.8), glycerol (48%), SDS (6%), beta-mercaptoethanol (6%), and bromophenol (0.03%). Cell lysates were denatured by heating before being applied to SDS-PAGE gel. After electrophoresis, proteins were transferred to nitrocellulose membranes, blocked for 1 h in blocking solution (1–5% BSA in TBST buffer), and incubated with specific primary antibodies overnight at 4°C. Primary antibodies were detected with HRP-conjugated secondary antibodies, and antibody-protein complexes were visualized using ECL (Pierce Biotech). Results are expressed as the ratio of target protein expression to that of an internal loading control, caveolin-1.

For immunofluorescence, 2.0 × 10^4^ cells were seeded onto 4-well chamber slides (Millipore) and subjected to the appropriate treatments as described in the relevant sections. Immunofluorescence assays were carried out according to standard methods as described by others [8, 15]. Briefly, cells were fixed with 1% paraformaldehyde in 0.1 M sodium phosphate buffer, pH 7.3 for 10 min at room temperature. Cells were washed in 1X PBS, permeabilized by incubating with 0.01% Tween-20/PBS for 10 min, and then washed again with 1X PBS. After the last wash, cells were blocked using blocking buffer (1% BSA, 2% normal serum, 0.1% Tween-20 in PBS) for 30 min. Blocking solution contained normal rabbit serum, the animal in which the primary antibody was generated. Once blocking was complete, the cells were incubated with specific primary antibodies overnight at 4°C. Bound antibody was visualized under the microscope (Nikon Ti-E Eclipse, Nikon Instruments, Melville, NY) by incubating for 1 h with secondary antibody labeled with Alexa Fluor 568 (TRITC). To visualize the nucleus, cells were exposed for 5 min at room temperature to a concentration of 300 nM DAPI (4′, 6-diamidino-2-phenylindole, dilactate) in PBS. DAPI was prepared and diluted based on manufacturer's instructions (Invitrogen). After washing, cells were mounted using ProLong Gold Antifade reagent (Invitrogen). Slides were sealed and allowed to dry overnight before imaging. Additionally, both positive and negative controls were prepared and imaged alongside the samples to correct for any background fluorescence and to serve as controls for quantitative analysis. Images were captured using an epifluorescence microscope (Nikon Instruments) with a 60x objective lens (NA 1.49) and an automated stage.

### 2.6. Glucose Uptake

Glucose uptake in myotubes was measured as previously described [11]. Briefly, myoblasts were plated in 6-well cell culture plates at a density of 2 × 10^5^ cells/well and allowed to differentiate under normal conditions. After differentiation, cells were washed with IX PBS and serum-starved in DMEM only for 3−5 h. Cells were stimulated with 100 nM insulin in DMEM for 30 min at 37°C. Each 6-well plate was setup so that wells 1, 2, and 3 did not contain insulin and wells 4, 5, and 6 did contain insulin. Insulin induction was stopped by washing the cells twice with 1 mL Krebs-Ringer HEPES (KRP) (121 mM NaCl, 4.9 mM KCl, 1.2 mM MgSO_4_, 0.33 mM CaCl_2_, 12 mM HEPES) minus glucose at room temperature. Cytochalasin B (5 *μ*L of 1 mM stock/1 mL cocktail) was used to normalize for nonspecific glucose uptake. Glucose uptake was determined after the addition of  ^3^H-2-deoxyglucose (1 *μ*L of 10 Ci/mmol stock/1 mL cocktail) in KRP buffer at 37°C for 5 min. Incorporation was terminated by washing the cells twice with 1 mL ice cold KRP plus 25 mM glucose. Cells were lysed on ice for 30 min with RIPA buffer. Following incubation, 0.5 mL cell lysates were mixed with 10 mL scintillation fluid (Scintiverse) and subjected to liquid scintillation counting. For protein quantitation (BCA method, Pierce Biotech), 250 *μ*L of the lysate was used and processed according to the manufacturer's instructions. Glucose uptake was expressed as disintegrations per minute per microgram of total protein (dpm/*μ*g). Data is reported as the glucose uptake stimulation expressed as the ratio of dpm/*μ*g of total protein in presence of insulin to that in the absence of insulin.

### 2.7. Densitometry Analysis

Relative densitometry analyses of the immunoblots were determined using Scion (http://scion-image.software.informer.com/) and Image J (http://rsb.info.nih.gov/ij/index.html) analysis software. By giving an arbitrary value of 1.0 to the respective control sample (caveolin-1) of each experiment, a ratio of relative density was calculated for each protein of interest.

### 2.8. Immunofluorescence Quantitation

Immunofluorescence images were analyzed using NIS-Elements software (AR v. 3.1) (Nikon Instruments). For each sample, 9 dual-channel images were captured and stitched together to form a large image (3 × 3). Mean fluorescence intensity per cell was calculated (MFI per cell = total image fluorescence/cell count). Cell count was determined using a nuclear stain (300 nM DAPI in PBS).

### 2.9. Statistical Analysis

The data are expressed as mean ± SE. For comparison of two groups, *P*  values were calculated using the two-tailed paired Student *t* test. In all cases *P* ≤ 0.05 was considered statistically significant, and *P* ≤ 0.10 was indicative of a trend.

## 3. Results and Discussion

To alleviate whole-animal complexities, C2C12, a mouse skeletal muscle cell line, was selected as an *in vitro *model to identify molecular and cellular targets of ATP10C and then assess its biological role, if any, in insulin signaling and glucose metabolism [16, 17].

Under permissive conditions, C2C12 myoblasts undergo differentiation to form myotubes. Differentiation of C2C12 myoblasts to myotubes was confirmed by visual inspection (see Supplemental Figure 1 in Supplementary Material available online at doi.10.1155/2012/152902), as well as by monitoring the expression of skeletal muscle-specific mRNAs for *MyoD* and *myogenin* [18]. Cells were collected on days 1, 3, and 5 during the differentiation process. Cells collected at day 1 represent the myoblasts, which are then stimulated to differentiate into myotubes by days 3–5. The activation of cell differentiation is characterized by the expression of myogenic regulatory factors including *MyoD* and *myogenin*. After proliferation, cells express *myogenin* which commits the myoblasts towards myogenic differentiation. This is followed by the expression of additional factors including but not limited to Myf-5 and MRF4 and permanent exit from the normal growth and proliferation cycles. As expected, the expression of *myogenin* increased as myoblasts were stimulated to differentiate (30% to 52%), and decreased when differentiation was complete (20%). Similarly, qPCR showed that *MyoD *expression increased dramatically on day 1 (172%), then steadily declined on days 3 and 5 (64% and 24%, resp.). Interestingly, *Atp10c* was expressed in both cell types, and its expression increased upon differentiation (42% to 64%) and steadily decreased as myotubes were formed (47% on day 5). Changes in *Atp10c* expression with differentiation were similar, but, not as striking as that of *myogenin* and *MyoD* (Supplemental Figure 2), ramifications of which are clearly beyond the scope of this study. The data thus demonstrated the expression of *Atp10c* mRNA in C2C12 cells, and gave us an opportunity to modulate its expression in both myoblasts and myotubes, both of which can have significant consequences in insulin signaling pathways.

One of the limitations of global gene-targeting and whole-animal approach is that adaptations over time might occur, possibly producing secondary phenotypes that are not directly linked to the mutation. In this case, the role of *Atp10c* could be shown either by generating knockout mice or using specific inhibitors against ATP10C. Since no inhibitors are available, and to avoid whole-animal complexities in transgenics, we modulated *Atp10c* expression *in vitro* in C2C12 cells by transiently transfecting them with three independent and commercially available *Atp10c*-specific siRNAs (Qiagen). ATP10C is a putative transmembrane domain protein, and as such a good antibody against ATP10C has yet to be generated, making experiments to study ATP10C challenging. Therefore, in this analysis, changes in *Atp10c* expression were determined solely at the mRNA level by quantitative PCR using QuantiTect primer assays (Qiagen). Out of the three siRNAs tested, only one, SI00906220, resulted in a significant knockdown of ≥70% (data not shown).

**Figure 1:**
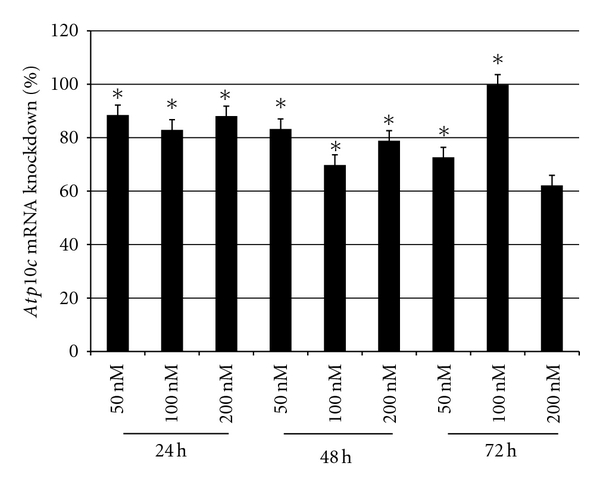
C2C12 cells were differentiated from myoblasts to myotubes as described in Section 2. Myotubes were transfected at each concentration of siRNA (SI00906220) (0, 50, 100, and 200 nM) collected at the above time points (24, 48, and 72 h). *Gapdh* (housekeeping gene) and *Atp10c* gene mRNA expression was analyzed using quantitative PCR. The percentage of knockdown was calculated at each concentration and time point based on the expression of the mock-transfected (0 nM) samples (*P < 0.7  based on Qiagen recommendations). Data represents three independent experiments with each sample repeated in triplicate.

As shown in Figure 1, significant knockdowns (70 to 100%) were observed in all samples with the exception of 200 nM, 72 h. Higher concentrations and longer time periods resulted in cell death and poor quality of cells (as judged visually). Taking these criteria into consideration as well as the optimization of concentration and time of transfection, 50 nM of siRNA (SI00906220) and a time point of 24 h were selected for all subsequent experiments. *Gapdh* was used as the housekeeping gene, and no significant mRNA changes in its corresponding expression in transfected cells were observed. Results thus suggest that changes in *Atp10c* expression after transfection with siRNA (SI00906220) were not an artifact, and there was no deleterious effect on C2C12 myotubes due to *Atp10c* silencing. All further experiments were carried out under these conditions in wild-type C2C12 (C2wt) and *Atp10c*-silenced C2C12 myotubes (C2*10c*/−) simultaneously. Since these transfections are transient, *Atp10c* mRNA knockdown was confirmed in each and every experiment.

**Figure 2:**
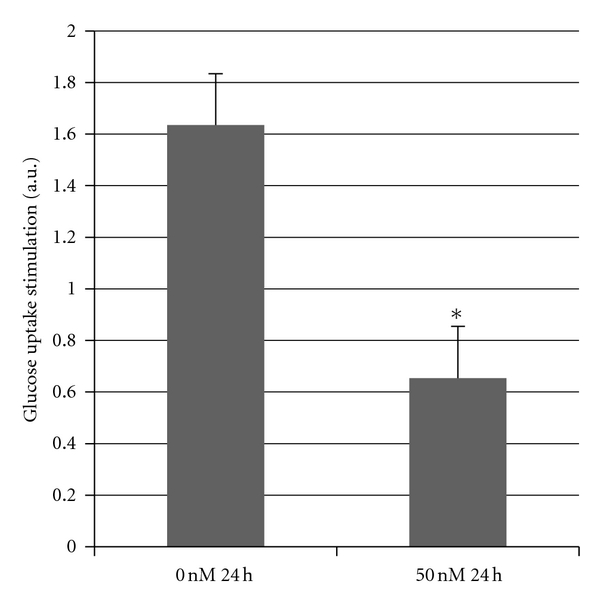
C2C12 cells were differentiated from myoblasts to myotubes as described in Section 2. Myotubes were transfected at each concentration of siRNA (SI00906220) (0 and 50 nM) at the designated time point (24 h). Cells were then stimulated with insulin (100 nM, 30 min), and a 2-DOG uptake was performed (*P < 0.05). Data is reported as the glucose uptake stimulation which is expressed as the ratio of dpm/*μ*g of total protein in presence of insulin to that in the absence of insulin.

Previous experiments in our laboratory have shown that on a high fat diet, there is a 35% decrease in glucose uptake in soleus muscle in *Atp10c *heterozygotes [5]. Based on this finding, we next determined whether downregulation of *Atp10c* expression had a similar effect *in vitro*. Glucose uptake was measured in C2wt and C2*10c*/− myotubes in basal (without insulin) and stimulated (100 nM insulin, 30 min) states. Fold change representing the glucose uptake stimulation/reduction between the C2wt and C2*10c*/− was compared. As shown in Figure 2, insulin stimulation caused a significant 2.54-fold decrease in 2-DOG uptake in C2*10c*/− cells (*P* < 0.05). Data thus complement the *in vivo* findings, suggesting that ATP10C is necessary for insulin-stimulated glucose uptake in skeletal muscle, and its knockdown renders the myotubes insulin resistant.

**Figure 3:**
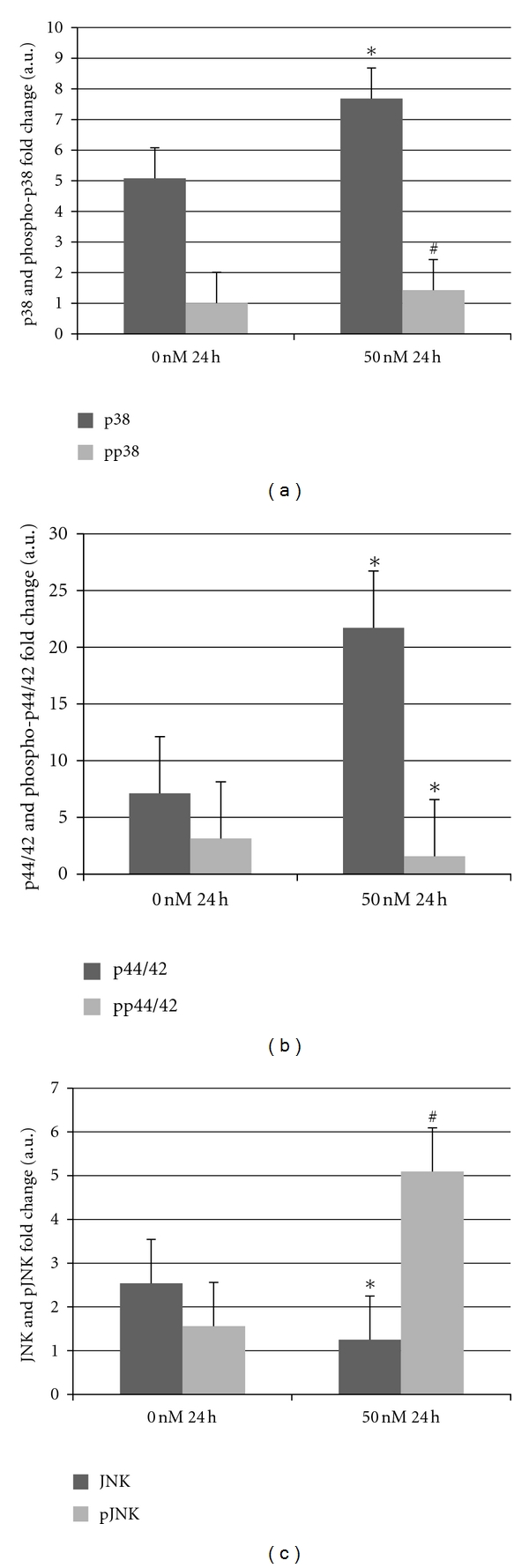
(a–c): C2C12 cells were differentiated from myoblasts to myotubes as described in Section 2. Myotubes were transfected at each concentration of siRNA (SI00906220) (0 and 50 nM) and collected at the designated time point (24 h). Proteins were collected from these samples and subjected to immunoblot analysis. Data shown are representative of multiple independent experiments (*n* = 2 to 4), all analyzed in triplicate (*P < 0.05, ^#^ *P* < 0.1).

Silencing *Atp10c* RNA decreases cellular glucose uptake, which might be of consequence to impaired insulin signaling. Insulin-induced glucose uptake into muscle and adipose tissue involves a series of intracellular signaling cascades, culminating in glucose disposal and metabolism [19–24]. The possible mechanisms include insulin-mediated activation of the insulin receptor and/or its downstream molecules, ultimately effecting GLUT4 expression and translocation. Since these processes are a complex interplay of a variety of proteins and because their changes have not been studied in *Atp10c* silencing, we sought to identify changes in the key proteins of one such signaling cascade, the MAPK pathway in the absence of any insulin stimulation. Specifically, in the present study, the effect of *Atp10c* silencing was considered on three essential MAPKs: p38 (MapK14), JNK, and p44/42, (ERK1/2). Total proteins isolated from C2wt and C2*10c*/− myotubes were subjected to a combination of immunoblot and immunofluorescence analysis (Figures 3(a)–3(c)).

**Figure 4:**
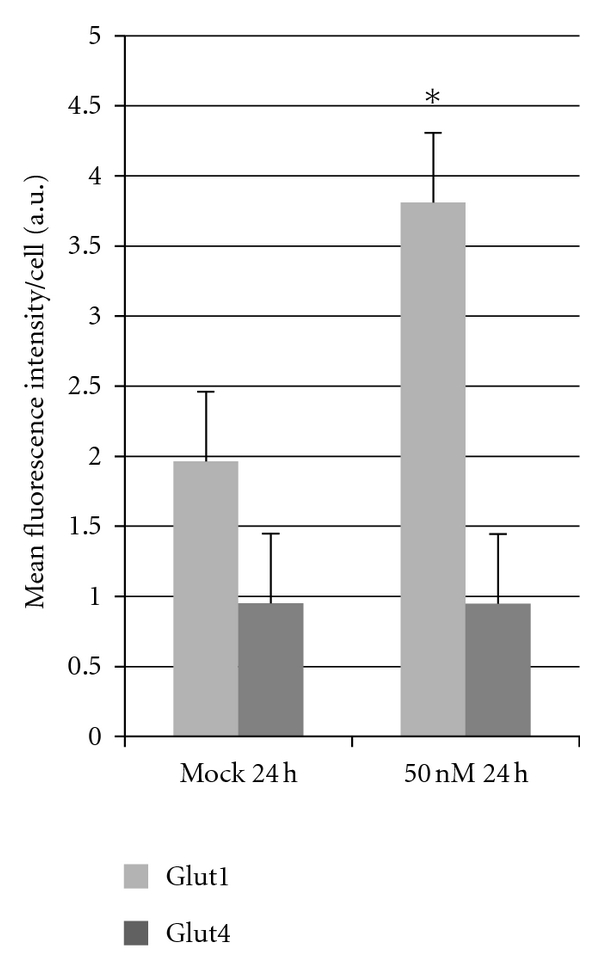
C2C12 cells were seeded onto chamber slides and allowed to differentiate from myoblasts to myotubes as described in the Materials and Methods section. Myotubes were then transfected at each concentration of siRNA (SI00906220) (0 and 50 nM) and collected at the designated time point (24 h). After transfections, cells underwent standard immunofluorescence processing and were imaged. Each sample was compared to negative and positive controls, which were used to quantify the image results (*P < 0.05).

For MAPK pathway analysis, results indicated significant upregulation of p38 (*P* = 0.02) and p44/42 (*P* = 0.04) and a significant downregulation of JNK (*P* = 0.001) and phospho-p44/42 (*P* = 0.05) in C2*10c*/− cells. While not significant, results indicated a trend for an increase in phospho-p38 (*P* = 0.1) (Figure 3(a)) and phospho-JNK (*P* = 0.06) (Figure 3(c)). Beside these signal transduction proteins, there was significant upregulation of MyoD (*P* = 0.01), Actin (*P* = 0.02) (Supplemental Figures 3(a) and 3(b)), and GLUT1 (*P* = 0.03). Most importantly, there was no significant change in GLUT4 expression (Figure 4).

The exact mechanism by which the MAPKs mediate glucose uptake is debatable. This is such a complex process that both *in vitro* and *in vivo* data are inconclusive. MAPKs, specifically, p38 and p44/42 proteins, regulate glucose uptake via insulin-dependent and independent pathways [25–27]. Our results suggest that when myotubes become insulin resistant by *Atp10c* silencing, as expected there is a significant increase in the expression of native MAPKs; however, they are not activated into their phosphorylated forms at a significant level. Data demonstrates that p38 and p44/42 are responsive to changes in *Atp10c* with increased expression, whereas there is no similar effect on JNK, suggesting that it is not a target at least in this pathway.

The importance of p38 in this process is further supported by the fact that there are significant changes in glucose transporter proteins as well. GLUT1 and GLUT4 have been demonstrated to be the key players of glucose clearance in peripheral tissues [5]. GLUT1 is responsible for glucose uptake in the basal state whereas GLUT4 is insulin responsive [28]. Defective uptake of glucose mediated by the GLUTs is a central feature of obesity and type 2 diabetes. Interestingly, microarray gene profiling of peripheral tissues obtained from *Atp10c* heterozygotes which were fed a high fat diet for 4 weeks demonstrated upregulation of p38 in soleus muscle [5].

Of relevance to our study, MAPK protein expression, specifically p38, has been shown to affect the expression of GLUT1 and GLUT4, which can subsequently affect glucose uptake in peripheral tissues [28]. The MAPK protein, p38, reportedly upregulates the expression of GLUT1, thereby altering glucose transport at the basal level, while also involved in an insulin-induced enhancement of intrinsic GLUT4 activity on the cell surface. Since, we do not see any increase in the basal glucose uptake, we strongly feel that the upregulation of GLUT1 is more of a compensatory mechanism against insulin resistance in C2*10c*/−, since there is no change in GLUT4; the precise underlying mechanism, however, remains to be clarified. This result may prove to be the most crucial, as GLUT4 is the main player in insulin-stimulated glucose metabolism. Reports additionally indicate that the activation of p38 reduces insulin responsiveness [25], so the exact mechanism by which the MAPK pathway is involved in glucose metabolism still remains controversial.

Up-regulation of MyoD and Actin in transfected cells (C2*10c/−*) suggests a regulatory mechanism by which the myotubes are trying to combat the stressful state of insulin resistance. By aiding with GLUT4-containing vesicle membrane movement and/or fusion, there is considerable evidence that Actin is essential for insulin-regulated glucose transport. Therefore, these cells may be overexpressing Actin acutely in preparation for insulin-stimulated glucose uptake [29].

**Figure 5:**
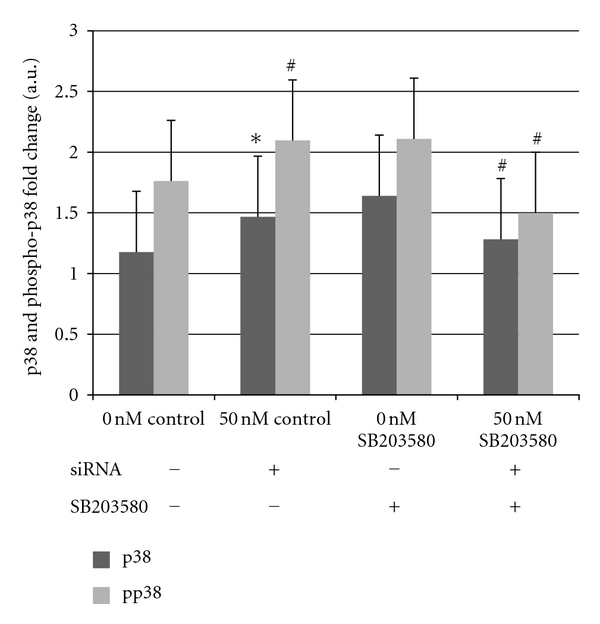
C2C12 cells were differentiated from myoblasts to myotubes as described in the Materials and Methods section. Myotubes were transfected at each concentration of siRNA (SI00906220) (0 and 50 nM) at the designated time point (24 h). Cells were then treated with the inhibitor SB203580 (10 nM) and collected after 60 min. Proteins were collected from these samples and subjected to immunoblot analysis (*P < 0.05, ^#^ *P* < 0.1).

To confirm the involvement of p38, the p38-specific inhibitor SB203580 was added to C2*10c*/− cells at a concentration of 10 *μ*m for 60 min [30, 31]. Protein samples were subjected to immunoblot analysis with p38 andphospho-p38 antibodies (Figure 5).

**Figure 6:**
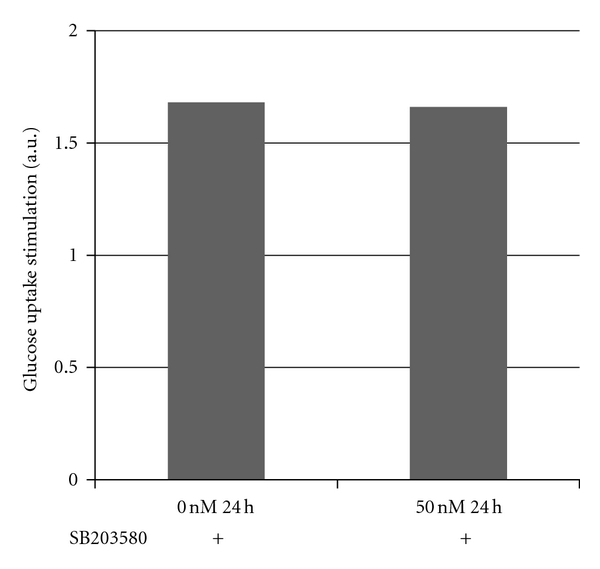
C2C12 cells were differentiated from myoblasts to myotubes as described in the Materials and Methods section. Myotubes were treated with the inhibitor SB203580 (10 nM) and collected after 60 min. Cells were then stimulated with insulin (100 nM, 30 min), and a 2-DOG uptake was performed (*P < 0.05, ^#^ *P* < 0.1).

Our results indicate that C2*10c*/− cells treated with 10 nM SB203580 for 60 min effected the expression of both p38 (*P* = 0.1) and phospho-p38 (*P* = 0.08) (Figure 5). While not significant, it appears that the inhibitor was able to partially restore the expression of all the proteins tested suggesting its action on the MAPKs. This was further confirmed by the glucose uptake assay performed on C2*10c*/− and MAPK-inhibited cells. Results from the assay showed that glucose uptake remained unchanged, confirming that the inhibitors are acting directly on the MAPK proteins and are not affected by changes in *Atp10c* expression (Figure 6).

## 4. Conclusions

A phenomenon known as phospholipid randomization affects the structure and function of many channels, transporters, and signal transducing proteins and has been implicated in several pathophysiology processes. Thus, maintaining the organization and activity of the lipid bilayer is essential for normal cell function. One class of proteins that performs this action is the flippases or type-IV ATPases [6]. In yeast studies, these proteins cause the translocation of glycerophospholipids, and this movement is necessary for intracellular membrane and protein trafficking [6]. *Atp10c* is one such phospholipid translocase which encodes for a type IV P-type ATPase. Our laboratory has demonstrated that *Atp10c *heterozygous mice are insulin resistant and have an altered insulin-stimulated response in peripheral tissues. Our obesity mouse model is dietinduced and shows insulin resistance characterized by hyperinsulinemia, hyperglycemia, hyperlipidemia, and obesity in association with glucose intolerance [2, 3, 11]. In fact, a recent publication has sited ATP10C as a potential biomarker for obesity and related metabolic disorders [32].

In the present study, we report, for the first time, a direct correlation between *Atp10c* mRNA expression levels and glucose metabolism, at least in part via the MAPK pathway in C2C12 skeletal muscle cells. Our results showed no significant change in the basal glucose uptake suggesting that when Atp10c is silenced using *Atp10c*-specific siRNA, an acute state of insulin resistance is observed. Conversely, cells are insulin sensitive when normal levels of *Atp10c* are maintained. The MAPK protein, specifically p38, is potentially influencing this system by exerting an effect on the glucose transporter proteins. Results from these experiments are interesting and lead to future experiments that will involve other signaling cascades (PI3K), insulin receptors, and substrates as well as other downstream factors, including GLUT4 translocation.

## Supplementary Material

The supplemental material for this manuscript contains three additional figures. Figure 1 Supplemental Material shows images of C2C12 myoblast differentiation from myoblasts into myotubes. The second figure, Figure 2 Supplemental Material, illustrates the mRNA expression of *MyoD*, *myogenin* and *Atp10c* genes in differentiating C2C12 cultured cells as analyzed using Real time PCR methods. Figure 3A-B Supplemental Material, the final figure presented, displays the protein expression of actin and MyoD after C2C12 myotubes were transfected at each concentration of siRNA (SI00906220) (0 nM and 50 nM) and collected at the designated time point (24 hours).
