# Supplementary material for: Transient Silencing of a Type IV P-Type ATPase, Atp10c, Results in Decreased Glucose Uptake in C2C12 Myotubes
Source: J Nutr Metab. 2012 Feb 29;2012:152902. doi: 10.1155/2012/152902 (PMC3317196; doi:10.1155/2012/152902)
Supplement: Supplementary file 1 — The supplemental material for this manuscript contains three additional figures. Figure 1 Supplemental Material shows images of C2C12 myoblast differentiation from myoblasts into myotubes. The second figure, Figure 2 Supplemental Material, illustrates the mRNA expression of MyoD, myogenin and Atp10c genes in differentiating C2C12 cultured cells as analyzed using Real time PCR methods. Figure 3A-B Supplemental Material, the final figure presented, displays the protein expression of actin and MyoD after C2C12 myotubes were transfected at each concentration of siRNA (SI00906220) (0 nM and 50 nM) and collected at the designated time point (24 hours). [file 152902.f1.pdf]

Hurst et al. Supplemental Material

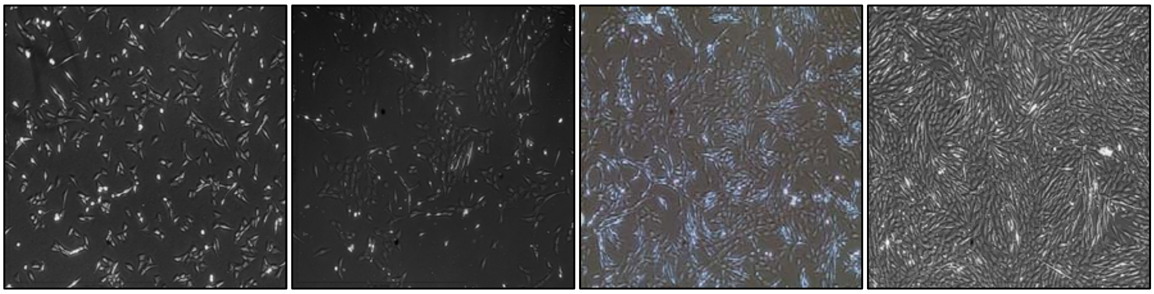

Figure 1 Supplemental Material: C2C12 myoblast differentiation into myotubes as described in the Materials and Methods section. Cells were seeded into 60 mm dishes and allowed to attach. After 24 hours, differentiation was stimulated via the addition of horse serum (2%) enriched media. Differentiation media was changed every 48 hours until the process was complete. Images were produced on Digital USB2 Microscope (Westover Scientific Inc., Mill Creek, WA) using Micron imaging software.
