# Supplementary material for: Transient Silencing of a Type IV P-Type ATPase, Atp10c, Results in Decreased Glucose Uptake in C2C12 Myotubes
Source: J Nutr Metab. 2012 Feb 29;2012:152902. doi: 10.1155/2012/152902 (PMC3317196; doi:10.1155/2012/152902)
Supplement: Supplementary file 2 [file 152902.f2.pdf]

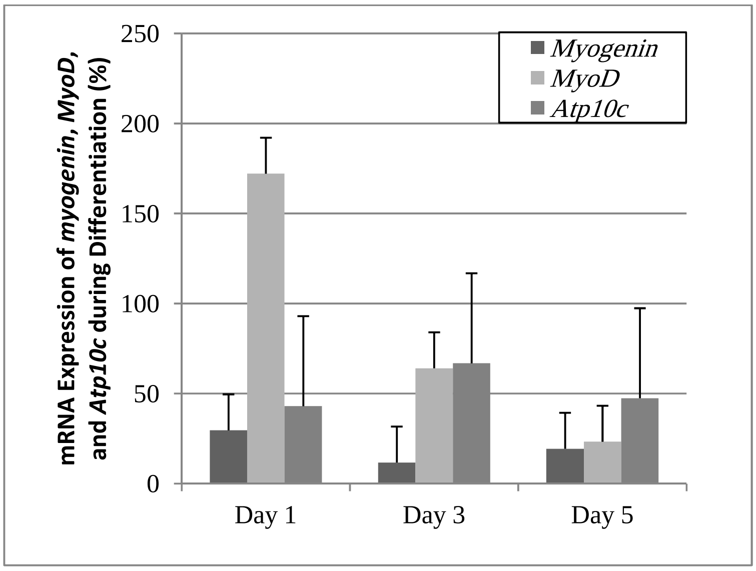

Figure 2 Supplemental Material: C2C12 cells were differentiated from myoblasts to myotubes as described in the Materials and Methods section. Cells were collected at the above time points and *Gapdh* (housekeeping gene), *MyoD, myogenin* and *Atp10c* gene mRNA expression was further analyzed using Real time PCR. Data represents three independent experiments with each sample repeated in triplicate.
