# Supplementary material for: Transient Silencing of a Type IV P-Type ATPase, Atp10c, Results in Decreased Glucose Uptake in C2C12 Myotubes
Source: J Nutr Metab. 2012 Feb 29;2012:152902. doi: 10.1155/2012/152902 (PMC3317196; doi:10.1155/2012/152902)
Supplement: Supplementary file 3 [file 152902.f3.pdf]

A.

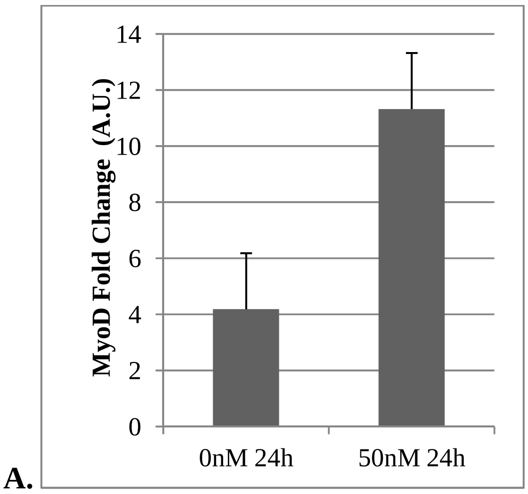

B.

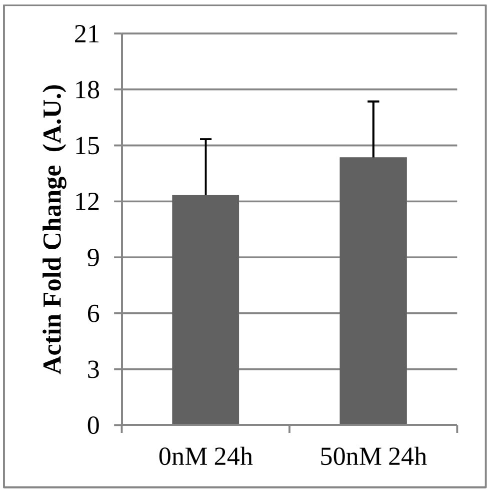

Figure 3A-B Supplemental Material: C2C12 cells were differentiated from myoblasts to myotubes as described in the Materials and Methods section. Myotubes were transfected at each concentration of siRNA (SI00906220) (0 nM and 50 nM) and collected at the designated time point (24 hours). Proteins were collected from these samples and subjected to immunoblot analysis. Data shown is representative of multiple independent experiments (n=2 to 4), all analyzed in triplicate (*p-value<0.05, # p-value<0.1).
